# 

**Published:** 1900-11

**Authors:** 


### CONTENTS.

ORIGINAL COMMUNICATIONS. PAGE

The Dentist and his Associates. By CHARLES OTIS KIMBALL, A.B., M.D., New York City . . . 709
Is Porcelain Inlay Work Impracticable? By DR. C. F. ALLAN . . . 717
The College Man in Dentistry. By HARRY L. GRANT, D.M.D., Providence, R. I. 721
Fungous Growth of the Pulp. By JULIO R. MARTINEZ, D.D.S. . . . 723
Fungoid Pulp. By WALTER W. BARTON, D.D.S. . . . 724
Fungoid Pulp. By R. L. GOODING, D.D.S. . . . 725
Professional Dignity. By J. MORGAN HOWE, New York . . . 727

ABSTRACTS AND TRANSLATIONS.

Errors caused by the False Interpretation of the Röntgen Rays, and their Medico-Legal Aspects. By CARL BECK, M.D., New York . . . 729

REPORTS OF SOCIETY MEETINGS.

The New York Institute of Stomatology . . . 738

Academy of Stomatology . . . 747

New Jersey State Dental Society . . . 756

EDITORIAL.

The New and the Old . . . 767

BIBLIOGRAPHY . . . 771

OBITUARY.

Manila Dental Society—Resolutions on the Death of Dr. Theodore Menges and Dr. George H. Cushing . . . 775

Dr. J. W. Clowes . . . 776

DOMESTIC CORRESPONDENCE.

On Methods of applying Silver Nitrate . . . 778

CURRENT NEWS.

Dental Commissioners of Connecticut . . . 779
Institute of Dental Pedagogics . . . 780

